# Vaginal microbiome in women from Greenland assessed by microscopy and quantitative PCR

**DOI:** 10.1186/1471-2334-13-480

**Published:** 2013-10-16

**Authors:** Raluca Datcu, Dionne Gesink, Gert Mulvad, Ruth Montgomery-Andersen, Elisabeth Rink, Anders Koch, Peter Ahrens, Jørgen Skov Jensen

**Affiliations:** 1 Microbiology and Infection Control, STI, Research and Development, Statens Serum Institut, Artillerivej 5, DK 2300, Copenhagen S, Denmark; 2 Dalla Lana School of Public Health, University of Toronto, Toronto, Ontario, Canada; 3 Centre for Primary Care, Nuuk, Greenland; 4 University of Greenland, Nuuk, Greenland; 5 Montana State University, Bozeman, Montana, USA; 6 Statens Serum Institut, Copenhagen, Denmark

**Keywords:** Bacterial vaginosis, Nugent criteria, Sensitivity, Specificity, ROC curve analysis, Clusters

## Abstract

**Background:**

Bacterial vaginosis (BV) is a common condition, although its aetiology remains unexplained. The aim of this study was to analyse the composition of vaginal microbiota in women from Greenland to provide a quantitative description and improve the understanding of BV.

**Methods:**

Self-collected vaginal smears and swabs were obtained from 177 women. The vaginal smears were graded for BV according to Nugent’s criteria. The vaginal swab samples were analysed by 19 quantitative PCRs (qPCRs) for selected vaginal bacteria and by PCR for four sexually transmitted infections (STIs).

**Results:**

STIs were common: *Mycoplasma genitalium* 12%, *Chlamydia trachomatis* 7%, *Neisseria gonorrhoeae* 1%, and *Trichomonas vaginalis* 0.5%. BV was found in 45% of women, but was not associated with individual STIs. Seven of the 19 vaginal bacteria (*Atopobium vaginae*, *Prevotella* spp., *Gardnerella vaginalis*, BVAB2, *Eggerthella*-like bacterium, *Leptotrichia amnionii*, and *Megasphaera* type 1) had areas under the receiver operating characteristic (ROC) curve > 85%, suggesting they are good predictors of BV according to Nugent. *Prevotella* spp. had the highest odds ratio for BV (OR 437; 95% CI 82–2779) in univariate analysis considering only specimens with a bacterial load above the threshold determined by ROC curve analysis as positive, as well as the highest adjusted odds ratio in multivariate logistic regression analysis (OR 4.4; 95% CI 1.4-13.5). BV could be subdivided into clusters dominated by a single or a few species together.

**Conclusions:**

BV by Nugent score was highly prevalent. Two of seven key species (*Prevotella* spp. and *A. vaginae*) remained significantly associated with BV in a multivariate model after adjusting for other bacterial species. *G. vaginalis* and *Prevotella* spp. defined the majority of BV clusters.

## Background

The human vaginal flora has an impact on the health of a woman and, if she is pregnant, on her foetus and newborn child. It is generally accepted that a healthy vaginal microbiome is dominated by *Lactobacillus* spp., while the replacement of these bacteria with facultative and strict anaerobes, such as *Gardnerella vaginalis*, *Prevotella* spp., *Peptostreptoccocus* spp., *Mobiluncus* spp., and a wide range of fastidious and uncultured bacteria leads to a switch from normal vaginal microbiota to bacterial vaginosis (BV) [1].

BV is the predominant cause of genital complaints in women of childbearing age worldwide [2]. About one in five adult women will have a BV episode annually; however, the condition is symptomatic in only half of women with BV. BV is associated with preterm birth and an increased risk of spontaneous miscarriage, increased risk of acquiring sexually transmitted infections (STIs), particularly genital herpes and HIV, as well as with post-abortion endometritis and post-hysterectomy vaginal cuff infection [1].

BV is commonly diagnosed clinically using Amsel’s criteria [3], which consist of four characteristics, including a homogeneous, white vaginal discharge; fishy odour of the vaginal discharge before or after addition of 10% potassium hydroxide; a pH of the vaginal fluid higher than 4.5; and the presence of clue-cells (squamous epithelial cells covered with adherent bacteria). At least three of the criteria must be present to diagnose BV. In research settings, a BV diagnosis is usually determined by microscopy of Gram-stained vaginal secretions, and the Nugent score is considered the gold standard [4]. More recently, an alternative scoring of vaginal smears, the Claeys’ criteria, was described [5], aiming to improve the discrimination between various morphotypes not accounted for in the Nugent score. The Claeys score is based on Ison-Hay’s criteria [6] with a more detailed evaluation of normal flora. Using the Claeys score, pregnant women with BV-like flora (grade 2 or 3), grade 1-like (small, short, irregularly shaped Gram positive rods) or 1-PMN (smears with over 50 polymorphonuclear leukocytes [PMN]/high power microscopy field in the absence of *Candida*) were shown to be at high risk for adverse pregnancy outcomes [7].

Despite significant research efforts in this area, no aetiological factors for BV have been identified [8]. Striking differences among populations have been observed; e.g., black women have significantly higher rates of BV than Caucasian women [9], and vaginal pH varies among different ethnic groups: Hispanic and black women have a higher average vaginal pH compared to Asians and Caucasians [10]. On the other hand, it has been suggested that a high Nugent score in asymptomatic women should be considered a separate group of normal flora (community group IV) [10]. Thus, the vaginal microbiota appears to depend partly on the ethnicity of the host and, consequently, of genetic and/or geographic and social factors.

It has been debated whether BV is sexually transmitted, as the carriage rates of *G. vaginalis*, a key bacterium in BV [11], were significantly higher in heterosexual men than among men who have sex with men [12], and BV was not found in sexually inexperienced women [13]. Many epidemiological features of BV are the same as those of STIs [14], although treatment of the male sexual partner does not decrease the rate of BV recurrence. However, BV may be considered a sexually enhanced disease, with the frequency of unprotected intercourse being a major predisposing factor [15].

Although the prevalence of STIs is high in the Greenlandic population [16,17], there are few publications describing the vaginal microbiota of women from Greenland. One study found a BV prevalence of 38% in Nuuk using Amsel’s criteria [18].

Microscopy of Gram-stained vaginal smears is considered the gold standard for diagnosing BV, but this approach does not provide detailed information about the vaginal microbiome, whereas species-specific real-time PCR can identify and quantify each bacterium. In this work, we present a cross-sectional, observational study on a cohort of women from Greenland aimed at presenting a detailed quantitative characterisation of the vaginal bacteria composition using microscopy and 19 quantitative real-time PCRs specific for bacteria that have been commonly reported as part of the human vaginal microbiome. We used threshold quantification to optimise the molecular techniques by using receiver operating characteristic (ROC) curve analysis to objectively define cut-off points for the diagnostic tests. We refer to this approach as quantitative detection, in which samples above the cut-off are considered positive, while samples with a bacterial load below the threshold are scored as negative. In qualitative detection, the samples are considered positive if DNA is detected (presence vs. absence). The diagnostic performance for BV detection was evaluated by both qualitative and quantitative detection using Nugent’s score as the reference.

## Methods

### Study population and specimens

A total of 196 women were included in “Inuulluataarneq” (the Greenland Sexual Health Project), a project aimed at quantifying the prevalence of STIs in Greenland and to identify individual, familial, social, cultural and environmental factors contributing to Greenland’s high STI rates [19,20]. The two inclusion periods were from July to October 2008 in Nuuk and November 2009 in Sisimiut during “Health Week.” Women were recruited from the general population via the population registry and by direct contact via telephone calls and advertising in local media, as previously described [17].

### Ethics statement

The project was approved by the Greenlandic ethics board, under the auspices of the Commission for Research in Greenland (KVUG), and the University of Toronto Research Ethics Board (21108 and 22173, September 2007 and June/October 2009, respectively). Study subjects provided written informed consent before participating in the study.

### Sample collection

Self-obtained vaginal swabs, vaginal smears, and first-void urine specimens were collected after an interviewer-administered structured survey. The women were given a pictorial instruction sheet on how to self-collect vaginal swabs by inserting a flocked swab (Copan, Brescia, Italy) approximately five cm into the vagina, using the breaking score as a guide to ensure that samples were collected properly. One swab was rolled onto a pre-labelled slide, which was left to air-dry. A second flocked swab was similarly obtained and placed in UTM transport medium (Universal Transport Medium, Copan, Brescia, Italy). First-void urine was collected in GeneLock nucleic acid stabilisation medium (Sierra Molecular Corp., Sonora, CA, USA). The samples were stored in a refrigerator at 5°C until they were shipped at weekly intervals from Nuuk at ambient temperature and from Sisimiut in one bulk shipment at the end of Health Week to Statens Serum Institut in Denmark.

### Microscopy

BV was diagnosed by microscopy of Gram-stained smears and classified by Nugent score [4]. All slides were scored by the same investigator (RD), who was blinded to any clinical or laboratory results.

Inflammation was graded in at least 5 microscopic fields as 0 (no PMN), + (< 10 PMN/ high power field [hpf]), ++ (10–50 PMN/hpf), or +++ (>50 PMN/hpf). A PMN count >10/hpf (i.e., ++ and +++) was considered abnormal [7]. The presence of *Candida* cells and hyphae was graded as: absence, few cells/hyphae (≤3 on average in at least 10 hpf) and many cells/hyphae (> 3 on average/hpf).

### DNA extraction and positive controls for PCR assays

Cells were released from vaginal swab specimens in UTM by thorough vortexing. Cells from 950 μl of the UTM were pelleted by centrifugation for 15 minutes at 30,000 × *g*. The supernatant was removed, and the pellet was vortexed for 60 seconds with 150 μl of 20% Chelex 100 slurry (BioRad, Hercules, CA, USA) in TE buffer (10 mM Tris–HCl [pH 8.0] and 1 mM EDTA). The samples were heated at 95°C for 10 minutes, and condensation droplets were collected by a brief centrifugation [21]. Urine specimens were treated similarly.

Positive controls and specificity controls for the PCRs were obtained by DNA extraction from culture collection strains of *Atopobium vaginae* (CCUG 38953^T^), *Sneathia sanguinegenes* (CCUG 41628^T^), *Leptotrichia amnionii* (DSM 16630), *Mobiluncus curtisii* (CCUG 21018^T^), *M. mulieris* (CCUG 20071^T^), *Lactobacillus iners* (CCUG 28746^T^), *L. crispatus* (CCUG 30722), *L. gasseri* (DSM 14869), *L. jensenii* (CCUG 21961), *Gardnerella vaginalis* (ATCC 3717^T^), *Mycoplasma hominis* (ATCC 15488)*, Ureaplasma parvum* (ATCC 27815 ^T^)*, U. urealyticum* (ATCC 27618 ^T^), *Finegoldia magna* (DSM 20470^T^), *Peptostreptococcus anaerobius* (CCUG 7835), and *Veillonella montpellierensis* (CCUG 48299), and from clinical isolates of *Prevotella oris*, *P. bivia* (2 different strains), *P. nigrescens* (2 different strains), *P. buccae* (2 different strains), *P. loeschii,* and *P. intermedia* identified by biochemical properties and MALDI-TOF and/or sequencing of 16S rRNA genes. DNA from cultured bacteria was extracted using the standard protocol with the QIAGEN Blood and Tissue kit (Qiagen, Hilden, Germany) except for *Lactobacillus spp*., for which the cells were suspended in 400 μl lysis buffer (20 mM Tris–HCl; 2 mM EDTA; 1.2% vol/vol Triton X-100) as previously published [22], 6 mg of lysozyme dissolved in 15 μl TE buffer was added and samples were incubated for 30 minutes at 37°C. Subsequently, the standard QIAamp DNA mini kit protocol was followed.

For uncultured bacteria BVAB 1, 2, 3, TM7, *Megasphaera* type 1 and 2, and *Eggerthella*-like bacterium, PCR products from a positive clinical sample were used as positive controls. The PCR products were gel-purified with a QIAquick Gel Extraction Kit (Qiagen) and DNA sequenced for verification.

Genomic and amplified DNA for positive controls were quantified fluorometrically with a Qubit fluorometer, using Quant-iT™ dsDNA HS Assay Kit (Life Technologies Corp., Paisly, UK). Standard curves for quantitative PCRs were generated using 10-fold dilutions ranging from 1 genome equivalent (geq)/μl to 10^7^ geq/μl in TE buffer containing 1 μg/ml of calf thymus DNA (D-8661; Sigma-Aldrich).

### PCR analyses

Vaginal swabs and FVU were examined by PCR for *Mycoplasma genitalium*, *Chlamydia trachomatis, Neisseria gonorrhoeae,* and *Trichomonas vaginalis* as previously described [17]. *M. genitalium* was detected by real-time PCR targeting the MgPa-gene [21] and confirmed by conventional PCR for the 16S rRNA gene [23]. *C. trachomatis* was detected by real-time PCR targeting the 16S rRNA gene [24] and all positive results were confirmed by real-time PCR for the *C. trachomatis* cryptic plasmid [24]. There was no evidence that the new variant of *C. trachomatis* was present in these samples [17]. *N. gonorrhoeae* was detected by a porA pseudogene real-time PCR [25]. *T. vaginalis* was detected by a conventional PCR with primers targeting a *T. vaginalis* specific repeat DNA fragment [26].

Quantitative PCRs were performed on vaginal swabs for selected BV associated bacteria, including *A. vaginae*, *S. sanguinegens*, *L. amnionii*, Bacterial Vaginosis-Associated Bacterium 1, 2, 3 and TM7 (BVAB 1, 2, 3 and TM7), *Megasphaera* type 1 and 2, *Eggerthella*-like bacterium, *M. curtisii* and *M. mulieris*, *L. iners*, *G. vaginalis, M. hominis, U. parvum, U. urealyticum, F. magna,* and *Prevotella* spp. Primers used for *Megasphaera* type 1 and *Eggerthella*-like bacterium as well as primers and probe for *G. vaginalis* were previously published by Fredricks [27,28], and specificity was documented by amplicon sequencing. For the remaining assays, specificity was documented by melt-curve analysis and during assay optimisation by gel-electrophoresis that demonstrated amplicons of the expected length. Sequencing (Big-Dye version 3.0, Life Technologies) of selected amplicons was used to document specificity.

PCR with TaqMan probes (*A. vaginae, S. sanguinegens, L. amnionii, L. iners, M. hominis*) was performed in a 50 μl final volume containing 1× PCR Reaction Buffer - 20 mM Tris–HCl pH 8.4, 50 mM KCl – (Life Technologies); 125 μM (each) of dATP, dCTP and dGTP; and 250 μM dUTP; 1 μM forward and reverse primers, 75 nM probe, 5 mM MgCl_2_, and 2 U Platinum^®^ Taq DNA polymerase (Life Technologies). For the *G. vaginalis* TaqMan probe assay, Platinum^®^ Quantitative PCR SuperMix-UDG (Life Technologies) and the same final concentrations of primers, probe and MgCl_2_ as above were used. *U. urealyticum* and *U. parvum* were detected in a multiplex PCR with 150 nM probes, 1 μM of the common forward primer and 2 μM of reverse primer for *U. urealyticum* and 1 μM of reverse primer for *U. parvum*; the other reagents were as previously described. An ABI 7500 Sequence Detection System was used with 96-well conventional blocks (Life Technologies) with a 95°C, 10 min initial denaturation and 50 cycles of 95°C for 15 s for denaturation and 60°C for 1 min for annealing and extension.

SYBR Green assays (BVAB1, BVAB2, BVAB3, BVABTM7, *Megasphaera* type 1 and 2, *Eggerthella*-like bacterium, *M. curtisii*, *M. mulieris*, *Prevotella* spp., and *F. magna*) were performed in a 50 μl final volume with reagents as described for TaqMan assays, except that the primers were used at 0.4 μM and MgCl_2_ concentrations varied between 1.5-3.5 mM, as listed in Additional file 1: Table S1. SYBR Green (Life Technologies) was used at a 1:5000 dilution. An ABI 7500 instrument was used with cycling conditions consisting of an initial 10 min denaturation followed by 10 cycles of touch-down PCR with a 1°C decrement per cycle until reaching the intended annealing temperature, which was used for the subsequent 30 cycles followed by a melt-curve analysis. Cycling conditions for each of the bacterial species are listed in Additional file 1: Table S1.

All results are expressed as the number of 16S rRNA gene copies/ml UTM.

All vaginal swab samples were checked for inhibition in the diagnostic PCRs detecting STI pathogens. However, to estimate the robustness and influence of non-target DNA on the SYBR Green assays (BVAB1, 2, 3, TM7, *Eggerthella*-like bacterium, *Megasphaera* type 1 and 2, *M. curtisii*, *M. mulieris*), the standard curves were evaluated with and without the presence of negative clinical samples.

### PCR method validation

Primers for *A. vaginae, L. amnionii, S. sanguinegens,* and *F. magna* were designed for the present study. The specificity of the primer sequences was examined by BLAST search of GenBank sequences. The TaqMan assays were challenged with 10^5^ genome equivalents (geq) of *A. vaginae, L. amnionii, and S. sanguinegens*. For the *F. magna* PCR, seven amplicons from random clinical specimens were sequenced and had 100% homology to the GenBank sequence; furthermore, purified DNA from the related species *Peptostreptococcus anaerobius* and *Veillonella montpellierensis* did not cross-react in the assay.

For BVAB1, BVAB2, *Megasphaera* type 2, BVAB TM7, *M. curtisii,* and *M. mulieris*, the published primers were slightly modified at the 5′-end to adjust the annealing temperature (see Additional file 1: Table S1). However, the 3′-end remained unchanged, and therefore the specificities of the primers as described in the original publications were preserved [27,28].

For BVAB3, a combination of forward and reverse primers published in two different studies were used and modified at the 5′-end (see Additional file 1: Table S1). A BLAST search showed a 100% match with the BVAB3 16S rRNA gene sequence and lacked any other significant matches. The sequencing of amplicons from a random clinical specimen revealed a 100% match with the published sequence, and the melt-curve analysis showed distinct peaks.

*M. curtisii* DNA did not cross react in the *M. mulieris* real-time assay and vice-versa.

For the *L. iners* assay*,* the forward and reverse primers were combined from assays described in two previous publications [22,29], modified at the 5′-end (see Additional file 1: Table S1) and used with a probe designed for the present study. Cross-reactions with *L. crispatus*, *L. gasseri,* and *L. jensenii* DNA were studied by challenging the assays with 10^5^ geq of each species*.*

The *Prevotella* spp. primers were previously published and slightly modified at the 5′-end (see Additional file 1: Table S1). The PCR primers were genus-wide and, according to a BLAST search, matched perfectly to 20 different *Prevotella* species: *P. timonensis, P. bivia, P. melaninogenica, P. buccae, P. histicola, P. disiens, P. buccalis, P. scopos, P. fusca, P. copri, P. salivae, P. oris, P. veroralis, P. paludivivens, P. pallens, P. oulorum, P. nigrescens, P. maculosa, P. corporis,* and *P. intermedia.* Dilutions ranging from 1 geq/5 μl to 10^4^ geq/5 μl were prepared from clinical isolates of *P. oris*, *P. bivia* (2 different strains), *P. nigrescens* (2 different strains), *P. buccae* (2 different strains), *P. loeschii,* and *P. intermedia* strains and used as templates in the *Prevotella* spp. assay to determine sensitivity for different strains.

### Precautions to avoid PCR product carryover

All pre-PCR manipulations were performed in a laboratory accredited according to the ISO 17025 standard to perform diagnostic PCR. Strict physical separation between PCR setup and analysis laboratories was maintained, and separate staff were dedicated to pre- and post-PCR work. All pre-PCR steps were carried out in laminar flow hoods, and sterile filter tips were used in all sample manipulations. All surfaces in the PCR setup laboratory were exposed to UV light between sessions and regularly wiped with a solution containing hypochlorite to destroy contaminating DNA [30]. At least two negative controls, including a mock-extracted sample, were included in each PCR run. All PCRs were performed with dUTP instead of dTTP to allow enzymatic prevention of PCR product carryover with uracil-*N*-glycosylase. Uracil-*N*-glycosylase was not used, however, because no carryover was observed.

### Determination of cut-off for optimal BV prediction

ROC curve analysis was used to determine the optimal bacterial load threshold in geq/ml UTM in women with and without BV, weighing sensitivity and specificity equally. For the calculation of the sensitivity and specificity for BV prediction, the Nugent score was used as the gold standard, and Grade II (intermediate flora) was excluded from the evaluation. For all 19 bacterial species, the sensitivity and specificity before ROC (qualitative detection) was calculated for 79 participants with BV and 73 participants without BV. Bacterial species with an area under the curve (AUC) > 85%, a value that suggested good discriminatory power, were selected for further analysis in a multivariate logistic regression model.

### Statistical methods

ROC curve analysis, Fisher’s exact test, and odd-ratios with confidence intervals as well as kappa statistics were calculated in StatsDirect version 2.7.8 (StatsDirect Ltd., Cheshire, UK).

Logistic regression analysis and Cochran-Armitage trend tests were performed in SAS version 9.2 (SAS Institute Inc. Cary, NC, USA). A heat-map was constructed by normalising the number of 16S rRNA gene copies/ml UTM (the proportion of gene-copies for an individual species to the total sum of gene-copies for all bacteria studied) and using the Heat map routine in the R program package version 2.15.0 (The R Foundation for Statistical Computing). Co-occurrence of bacterial taxa was investigated by calculating Spearman correlation coefficients using the routine correlation orders in the R program package. The significance level was 5%, and two-sided results were used throughout.

## Results

### Study population

Out of a total of 196 women enrolled in “Inuulluataarneq,” 177 women provided both swabs and suitable smears and were considered eligible. The women ranged from 15 – 65 years in age, with a median of 24 and mean of 29 (Table 1). Six participants were between 55 and 65 years old and presumably post-menopausal, but no information regarding age of menopause was collected. The median lifetime number of male sexual partners was 12.5, range 0 – 320 and interquartile range 20. Among the 168 women who provided information about their lifetime number of female sexual partners, 30 women had female sexual partners, including 26 who had male partners as well. Twelve women were registered as having no history of male or female sex partners, of whom four were diagnosed with BV by Nugent’s classification. The majority of the women (72%, 117 of 163 who answered the question) reported that they would always use a condom with a new partner they had only known for a few weeks. Participants were asked whether they had any STI symptoms and 67 (75%) of the 89 women who answered this question declared that they had no symptoms, 15 women said they did not know and 7 women reported symptoms. Among the women who reported symptoms, two had STIs and four had BV. No questions were asked about contraceptive use, douching or recent antibiotic use. However, douching is known not to be a common practice in Greenland.

**Table 1:** Study participants, characteristics and demographics


**Age**	
Range	15-65 years
Median (50th percentile)	24 years
Lower quartile (25th percentile)	19
Upper quartile (75th percentile)	37
**Inclusion town**	**N**^1 ^**(%)**
Nuuk	83 (47%)
Sisimiut	94 (53%)
**BV diagnosis by Nugent score**	**N (%)**
Grade I (normal)	73 (41%)
Grade II (intermediate)	25 (14%)
Grade III (BV)	79 (45%)
**STIs**^2 ^**and yeast infection**	**N (%)**
*Mycoplasma genitalium*	22 (12%)
*Chlamydia trachomatis*	12 (7%)
*Neisseria gonorrhoeae*	2 (1%)
*Trichomonas vaginalis*	1 (0.5%)
Yeasts^3^	14 (8%)
**Lifetime number of male sexual partners**^4^	
Range	0-320
Median (50th percentile)	12.5
Lower quartile (25th percentile)	5
Upper quartile (75th percentile)	25
**Lifetime number of female sexual partners**^4^	
Range	0-10
Median (50th percentile)	0
Lower quartile (25th percentile)	0
Upper quartile (75th percentile)	0

^1^N indicates number of participants.

^2^Considered positive if specific PCR was found positive in swab, urine or both.

^3^Either spores, hyphae or both by microscopy.

^4^Only 168 women gave information.

As previously described [17], 22 women (12%) were positive for *M. genitalium*, 12 (7%) were positive for *C. trachomatis*, 2 (1%) for *N. gonorrhoeae* and one woman had *T. vaginalis* (0.5%) in either the vaginal swab sample or in the first void urine. Three women had a mixed infection with *M. genitalium* and *C. trachomatis.*

### PCR methods validation

No cross-reactions were observed in the *A. vaginae, L. amnionii* or *S. sanguinegens* TaqMan assays when challenged with 10^5^ geq of the other species. Similarly, no cross reactions with *L. crispatus*, *L. gasseri* or *L. jensenii* DNA were observed in the *L. iners* assay*.*

The sensitivity of the *Prevotella* spp. assay for various species was determined to be 1 geq for *P. oris,* 10 geq and 100 geq for two strains of *P. bivia*, 10 geq for *P. nigrescens* (two strains), 1 geq for *P. buccae* (two strains), and 80 geq for *P. intermedia*. For *P. loeschii*, the sensitivity of the *Prevotella* spp. assay was much lower (4x10^3^ geq), as there were several mismatches in the primer sequence.

The limit of detection for the real-time PCR assays is shown in Additional file 1: Table S1.

### BV microscopy results

The BV prevalence was 45% as determined by Nugent’s classification, and 73 participants (41%) had a normal score, while 25 (14%) had an intermediate score (Table 1). Most women (80%) had normal leucocyte counts, i.e., below 10 PMN/hpf, while 35 women (20%) had more than 10 PMN/hpf, suggesting inflammation. Among the women with high leucocyte counts, 11 (31%) were positive for STIs, and 2 had vulvovaginal candidiasis. Fourteen women (8%) carried yeasts, as determined by microscopy (Table 1).

### Determination of cut-off for optimal BV prediction

Because the sensitivity and specificity of BV prediction by qualitative detection (presence versus absence) were quite low for all species (Table 2), ROC curve analysis was used to determine the optimal threshold for the bacterial load (cut-off) for BV prediction. The cut-off or threshold according to ROC curve analysis is shown for each species in Table 2. An area under the curve (AUC) of > 85%, suggesting a good discriminatory power, was found for *A. vaginae* (97%)*, Prevotella* spp. (96%), *G. vaginalis* (95%), BVAB2 (94%), *Eggerthella*-like bacterium (91%), *L. amnionii* (89%), and *Megasphaera* type 1 (88%). The ROC curve for *L. iners* was concave because this bacterium was predictive for normal vaginal flora.

**Table 2:** **Sensitivity and specificity before (qualitative detection**^**1**^**) and after (quantitative detection**^**2**^**) applying ROC curve analysis**

**Species**	**Qualitative detection**^**1**^				**Quantitative detection**^**2**^					**AUC**	
	**Se (%)**	**95% CI**	**Sp (%)**	**95% CI**	**Se (%)**	**95% CI**	**Sp (%)**	**95% CI**	**Cut-off/ml**	**Size (%)**	**95% CI**
Atopobium vaginae*	100	95-100	25	15-36	95	88-99	92	83-97	≥55556	97	95-99
*Sneathia sanguinegens*	75	64-84	86	76- 93	67	56-77	97	90-100	≥14127	85	79-90
Leptotrichia amnionii*	86	76-93	59	47- 70	80	69-88	97	90-100	≥1746	89	83-94
BVAB 1	67	56-77	68	57-79	54	43-66	93	85-98	≥9841	75	68-82
BVAB 2*	91	83-96	79	68-88	87	78-94	97	90-100	≥3492	94	90-98
BVAB 3	66	54-76	86	76-93	66	54-76	92	83-97	≥95	80	74-86
BVAB-TM7	34	24-46	100	95-100	34	24-46	100	95-100	≥2698	67	62-72
*Megasphaera *type 1*	86	76-93	59	47-70	81	71-89	99	93-100	≥41111	88	82-94
*Megasphaera *type 2	32	22- 43	93	85-98	28	18-39	100	95-100	≥1111	63	58-69
*Eggerthella*-like bacterium*	95	88-99	36	25-48	78	68-87	96	88-99	≥297937	91	86-96
*Mobiluncus curtisii*	25	16-36	99	93-100	25	16-36	99	93-100	≥794	62	57-67
*Mobiluncus mulieris*	20	12-31	93	85- 98	15	8-25	99	93-100	≥166349	57	52-62
Gardnerella vaginalis*	100	95-100	3	0.3-10	87	78-94	92	83-97	≥4803175	95	91-98
*Mycoplasma hominis*	63	52-74	85	75-92	58	47-69	96	88-99	≥3968	78	71-84
*Ureaplasma parvum*	51	39-62	49	37- 61	28	18-39	75	64-85	≥31746	50	42-59
*Ureaplasma urealyticum*	18	10-28	90	81-96	14	7-24	95	87-98	≥22381	54	49-59
*Finegoldia magna*	63	52-74	59	47-70	63	52-74	59	47-70	≥159	61	52-69
*Prevotella *spp.*	97	91-100	4	0.8-12	95	88-99	96	88-99	≥888889	96	92-100

^1^Presence versus absence as determined by qPCR.

^2^Presence (≥ cut-off/ml) versus absence (< cut-off/ml) with cut-off determined by ROC curve analysis.

A total of 79 participants with Nugent III and 73 with Nugent I were used for the evaluation. Intermediate flora not included. *L. iners* is excluded from the table because it acts as a negative predictor for BV.

*Bacterial species with area under the ROC curve (AUC) > 85%.

*A. vaginae* had the largest AUC (97%), indicating the best diagnostic accuracy. A combination of *A. vaginae* and *Prevotella* spp. in quantitative detection had a specificity of 99% and sensitivity of 90%, while *A. vaginae* or *Prevotella* spp. gave optimal sensitivity (100%) but a specificity of 89%.

### Relationship between PCR results and microscopy

The average number of non-*Lactobacillus* species detected per participant was 11.7 (range 5–16) for women with BV, 6.2 (range 3–10) for women with a normal score, and 8.6 (range 4–15) for women with an intermediate score using qualitative data (presence versus absence) from the 18 specific assays. For each group, the difference in diversity was statistically significant compared to any other group.

The percentage of cases in which each qPCR assay was positive by Nugent score category is presented in Table 3. A significantly increasing trend in the proportion of positive results in qualitative detection was found with increasing BV grade for all non-*Lactobacillus* species except *G. vaginalis, U. parvum, U. urealyticum,* and *Prevotella* spp., while all non-*Lactobacillus* species except *U. parvum* and *U. urealyticum* showed a significantly increasing trend in quantitative detection. The bacterial load of all non-*Lactobacillus* species increased statistically significantly with the progression from normal to BV microbiota for all species studied except *U. parvum* and *U. urealyticum*. The median bacterial loads for all species were higher for BV than for the normal microbiome except BVAB TM7, *Megasphaera* type 2, *M. curtisii, M. mulieris,* and *U. urealyticum,* for which medians for both Nugent I and III were 0*.* For *U. parvum* and *L. iners*, medians for Nugent I were higher than those for Nugent III.

**Table 3:** **Proportion of women in which non-*Lactobacillus *species were detected**

	**Nugent I (0–3)**		**Nugent II (4–6)**		**Nugent III (7–10)**		**p-values for trend**	
	**N = 73**		**N = 25**		**N**^**3**^ **= 79**			
	**Qualitative**^**1 **^**detection**	**Quantitative**^**2 **^**detection**	**Qualitative**^**1 **^**detection**	**Quantitative**^**2 **^**detection**	**Qualitative**^**1 **^**detection**	**Quantitative**^**2 **^**detection**	**Qualitative detection**	**Quantitative detection**
	**N (%)**	**N (%)**	**N (%)**	**N (%)**	**N (%)**	**N (%)**	**p-value**	**p-value**
*A. vaginae*	55 (75)	6 (8)	22 (88)	13 (52)	79 (100)	75 (95)	<0.0001	<0.0001
*S. sanguinegens*	10 (14)	2 (3)	9 (36)	5 (20)	59 (75)	53 (67)	<0.0001	<0.0001
*L. amnionii*	30 (41)	2 (3)	15 (60)	6 (24)	68 (86)	63 (80)	<0.0001	<0.0001
BVAB 1	23 (31)	5 (7)	11 (44)	6 (24)	53 (67)	43(54)	<0.0001	<0.0001
BVAB 2	15 (21)	2 (3)	10 (40)	8 (32)	72 (91)	69 (87)	<0.0001	<0.0001
BVAB 3	10 (14)	6 (8)	11 (44)	9 (36)	52 (66)	52 (66)	<0.0001	<0.0001
BVAB-TM7	0 (0)	0 (0)	6 (24)	6 (24)	27 (34)	27 (34)	<0.0001	<0.0001
*Megasphaera* type 1	30 (41)	1 (1)	17 (68)	9 (36)	68 (86)	64 (81)	<0.0001	<0.0001
*Megasphaera* type 2	5 (7)	0 (0)	3 (12)	2 (8)	25 (32)	22 (28)	<0.0001	<0.0001
*Eggerthella*-like bacterium	47 (64)	3 (4)	18 (72)	6 (24)	75 (95)	62 (78)	<0.0001	<0.0001
*M. curtisii*	1 (1)	1 (1)	3 (12)	3 (12)	20 (25)	20 (25)	<0.0001	<0.0001
*M. mulieris*	5 (7)	1 (1)	4 (16)	3 (12)	16 (20)	12 (15)	0.018	0.003
*G. vaginalis*	71 (97)	6 (8)	24 (96)	10 (40)	79 (100)	69 (87)	0.19	<0.0001
*M. hominis*	11 (15)	3 (4)	7 (28)	4 (16)	50 (63)	46 (58)	<0.0001	<0.0001
*U. parvum*	37 (51)	18 (25)	10 (40)	5 (20)	40 (51)	22 (28)	0.99	0.64
*U. urealyticum*	7 (10)	4 (5)	4 (16)	2 (8)	14 (18)	11 (14)	0.15	0.08
*F. magna*	30 (41)	30 (41)	17 (68)	17 (68)	50 (63)	50 (63)	0.006	0.006
*Prevotella spp.*	70 (96)	3 (4)	24 (96)	12 (48)	77 (97)	75 (95)	0.59	<0.0001

^1^Presence versus absence as determined by the target qPCR.

^2^Presence (≥ cut-off/ml) versus absence (< cut-off/ml) with cut-off determined by ROC curve analysis.

^3^ N indicates number of participants.

### Associations between age, lifetime number of male sexual partners, STIs and BV

The median age was the same (24 years) for women with Nugent I flora and women with Nugent III (p = 0.8); no relationship between the lifetime number of male sexual partners and BV could be found.

No statistically significant relationships between individual STIs and BV could be found.

### Associations between different vaginal bacteria and BV

In the qualitative detection, all bacteria were significantly associated with BV except for *G. vaginalis, Prevotella spp., U. parvum, U. urealyticum,* and *L. iners*. The strength of association between BV and the bacteria under study increased when odds-ratios were calculated using quantitative detection, i.e., with cut-offs determined by ROC curve analysis (Table 4). For *A. vaginae*, *G. vaginalis,* and BVAB TM7, the OR could not be calculated for qualitative detection due to the presence of these bacteria in all women with BV or absence in all women without BV (BVAB TM7). The OR for *L. iners* was 0 in qualitative detection. In quantitative detection, the OR could not be calculated for BVAB TM7 and *Megasphaera* type 2 due to their absence in women without BV or, in the case of *L. iners*, presence in all women without BV. *U. parvum*, *U. urealyticum,* and *L. iners* were not associated with BV. With quantitative detection and cut-off determined by ROC, all other bacteria were statistically associated with BV (p < 0.01).

**Table 4:** Odds-ratios (OR) and 95% confidence intervals (CI) (Fisher’s exact test) for BV

**Species**	**Qualitative detection**			**Quantitative detection**		
	**Observed OR**	**95% CI**	**p-value**	**Observed OR**	**95% CI**	**p-value**
*Atopobium vaginae*	∞	6-∞	< 0.0001	209	50-985	<0.0001
*Sneathia sanguinegens*	19	8-48	< 0.0001	72	16-634	< 0.0001
*Leptotrichia amnionii*	9	4-21	< 0.0001	140	30-1233	< 0.0001
BVAB 1	4	2-9	< 0.0001	16	6-56	< 0.0001
BVAB 2	40	14-120	< 0.0001	245	49-2191	< 0.0001
BVAB 3	12	5-30	< 0.0001	22	8-67	< 0.0001
BVAB-TM7	∞	9-∞	< 0.0001	∞	9-∞	< 0.0001
*Megasphaera* type 1	9	4-21	< 0.0001	307	43-12480	< 0.0001
*Megasphaera* type 2	6	2-22	0.0002	∞	7-∞	< 0.0001
*Eggerthella*-like bacterium	10	3-43	< 0.0001	85	23-453	< 0.0001
*Mobiluncus curtisii*	24	4-1025	< 0.0001	24	4-1025	< 0.0001
*Mobiluncus mulieris*	3	1-13	0.019	13	2-559	0.002
*Gardnerella vaginalis*	∞	0.2-∞	0.229	77	24-263	< 0.0001
*Lactobacillus iners*	0	0-2	0.121	ND	ND	ND
*Mycoplasma hominis*	10	4-23	< 0.0001	33	9-171	< 0.0001
*Ureaplasma parvum*	1	0.5-2	>0.9999	1	0.5-3	0.7
*Ureaplasma urealyticum*	2	0.7-6	0.1651	3	0.7-13	0.104
*Finegoldia magna*	2	1-5	0.0091	2	1-5	0.0091
*Prevotella spp.*	2	0.2-20	0.671	437	82-2779	< 0.0001

Nugent grade I (73 women) compared to grade III (79 women). Intermediate flora not included.

Detection of each bacterial species is after qualitative or quantitative detection with cut-off determined by ROC curve analysis.

ND indicates not determined.

### Best prediction of BV in a multivariate logistic regression model

When analysing the seven bacteria with the largest AUCs (marked with stars in Table 4) in a multivariate logistic regression model, only *Prevotella* spp. and *A. vaginae* remained associated with BV, with p-values of 0.0095 and 0.03 and odds ratios of 4.4 and 1.9, respectively (Table 5), after adjusting for other bacteria.

**Table 5:** Adjusted odds ratios with 95% confidence intervals and p-values in a multivariate logistic regression analysis

**Species**	**Logistic regression model**		
	**Adjusted OR**	**95% CI**	**p-value**
*Atopobium vaginae*	1.9	1.1-3.6	0.03
*Leptotrichia amnionii*	1.1	0.6- 2.2	0.7
*BVAB2*	2.0	0.9-4.4	0.07
*Megasphaera* type 1	1.4	0.9-2.4	0.1
*Eggerthella*-like bacterium	1.1	0.6-2.0	0.6
*Gardnerella vaginalis*	1.6	0.7- 3.9	0.2
*Prevotella* spp.	4.4	1.4-13.5	0.0095

The seven bacterial species were selected by ROC curve analysis with an area under the curve >85%.

Data are log_10_ of 16S rRNA gene copies plus one and outcome is BV compared with normal flora as determined by Nugent’s score.

### Vaginal bacterial communities in women with and without BV

**Figure 1:**
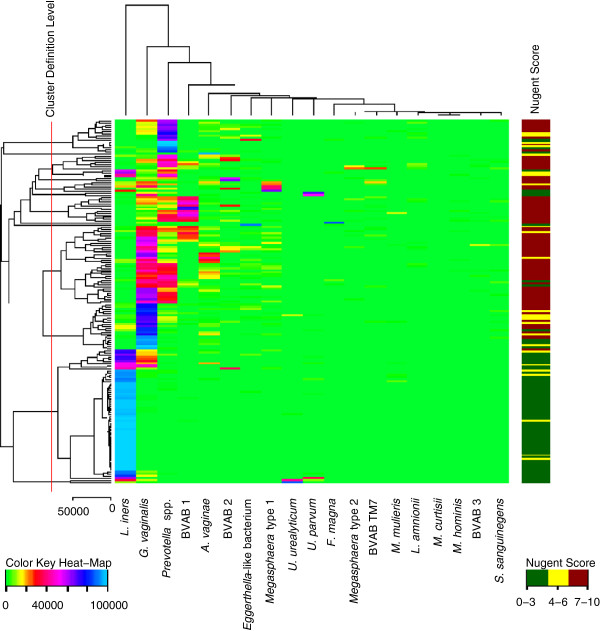
**Vaginal bacterial communities.** Heat-map constructed by normalising the number of 16S rRNA gene copies/ml UTM for vaginal bacteria in 177 women from Greenland with or without BV. Nugent scores are shown in the vertical column. The scale bar represents the Euclidean distance, corresponds to 50,000 and is calculated after Pythagorean metric. The colour key shows colour code and corresponds to normalised geq/ml UTM for each bacterium. The vertical red line indicates the level that was chosen for defining the clusters.

The vaginal bacterial community composition as shown by a clustering tree attached to a heat-map of normalised values of 16S rRNA gene copies was diverse and heterogeneous in women with BV. *L. iners* was the main species detected in women without BV (Figure 1). Among women with BV, three clusters were dominated by single taxa, i.e., *Prevotella* spp., BVAB1, and *G. vaginalis*; one cluster was dominated by *G. vaginalis*/*Prevotella* spp. while another cluster had BVAB1/*G. vaginalis* (Figure 1). The remaining BV clusters contained multiple bacterial species. Among the studied bacteria, *L. iners* was the best represented species among women without BV, while *Prevotella* spp. and *G. vaginalis* were present in higher proportions than the rest of bacteria were in women with Nugent III.

**Figure 2:**
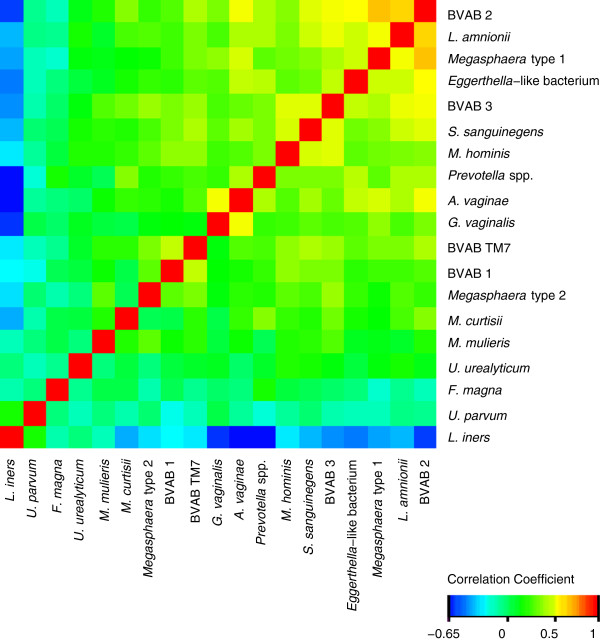
**Co-occurrence of bacterial species.** Hierarchically clustered Spearman correlation coefficients between bacterial species. Correlation values range from -0.65 to 1. Diagonal elements have a correlation of 1. Several bacteria associated with BV occur together. A tabulation of the coefficients is available in (Additional file 2: Table S2).

Co-occurrence of bacterial taxa was investigated by calculating Spearman correlation coefficients (Figure 2, see Additional file 2: Table S2 for a tabulation of the coefficients). The most significant cluster of correlations above 0.5 was found between BVAB2 and *A. vaginae*, *S. sanguinegens*, *L. amnionii*, BVAB3, *Megasphaera* type 1, and *Eggerthella*. Strong correlations were also observed for *L. amnionii* with BVAB2 and 3, *Megasphaera* type 1, and *Eggerthella*. Significant interactions were also found for *G. vaginalis* with *A. vaginae* and for *M. hominis* with *S. sanguinegens* and BVAB3. Negative correlations were found primarily for *L. iners*, which had correlations below -0.5 with *A. vaginae, Prevotella* spp., BVAB2, and *G. vaginalis.*

## Discussion

We found a high prevalence of BV in women from Greenland, with 45% of the women scoring positive for BV as determined by Nugent score. These figures are slightly higher than the 38% found in STD clinic attendees in 1991 [18] and comparable with the prevalence of BV in African women [31]. STIs are highly prevalent in Greenland, with chlamydia rates above 5,000/100,000 inhabitants. We also found *N. gonorrhoeae* in 1%, *C. trachomatis* in 7%, and *M. genitalium* in 12% of women tested, but *T. vaginalis* in only one woman (0.5%). BV has been associated with *C. trachomatis*[32] and *N. gonorrhoeae* infections [33-35], but we did not find a statistically significant association between any of the STIs and BV.

In contrast to other studies [36,37], no difference in age between women with and without BV could be found. An association between the lifetime number of sexual partners and/or a recent new partner has been found in several studies [14]; however, we did not confirm this association in the present study. Whether this outcome can be explained by the high number of male partners reported by the women in the present study is not clear. The women in this study reported a median of 12.5 partners (Table 1), much higher than the median of four partners reported by women in the UK National Survey of Sexual Attitudes and Lifestyles (NATSAL-2000) [38]. Thus, it is possible that the effect of the number of partners was already saturated in the current study’s population.

*L. iners* was detected by PCR in every participant with normal flora at a median concentration as high as 3 × 10^6^ 16S rRNA copies/ml UTM, a value higher than in women with BV (median 8 × 10^5^ 16S rRNA copies/ml). This finding is in accordance with other studies that suggest *L. iners* is present in the majority of women regardless of BV status [27]. Unfortunately, our DNA extraction method did not allow for reproducible quantitative detection of other *Lactobacillus* spp., and the amount of the original specimen did not allow for a repeated extraction. The incomplete lysis of *Lactobacillus* spp. other than *L. iners* was confirmed in an experiment comparing the Chelex extraction used in this study with a modified QIAamp extraction protocol that included a pre-incubation step with lysozyme on clinical specimens collected independently from the present study. *L. iners* was detected with good agreement between the two methods, but 11 of 12 *L. gasseri* positive specimens showed false negatives after Chelex extraction. Therefore, only *L. iners* qPCR was performed on the samples in this study, and consequently, we could not classify the flora according to community groups, as previously described by Ravel *et al. *[10].

In the present study, we showed that PCR may be a useful tool in diagnosing BV. However, we found that most of the BV-associated bacteria were also present in women without BV, and therefore, qualitative detection could not be used for diagnostic purposes. Consequently, we used quantitative data obtained by applying ROC curve analysis to determine the optimal cut-off level for diagnostic purposes. It was found that seven species (*A. vaginae*, *Prevotella* spp., *G. vaginalis*, BVAB2, *Eggerthella*-like bacterium, *L. amnionii*, and *Megasphaera* type 1) showed promising diagnostic potential in that they had areas under the ROC curve of > 85%, which suggests good discriminatory power for BV prediction. Some of these species, i.e., *A. vaginae*, *G. vaginalis*, BVAB2, and *Megasphaera* type 1, have been previously reported to be useful predictors for BV [39,40]. The same set of qPCRs applied on vaginal swabs from a Swedish population revealed the same seven bacterial species as the best predictors for BV [41]. However, the thresholds for bacterial load in our study were not identical to the study by Shipitsyna. This discrepancy can be explained to some extent by the use of different methods for diagnosing BV (Nugent in the present study in contrast to Amsel’s criteria in the study by Shipitsyna), different DNA extraction methods (simple lysis by boiling with Chelex resin versus a Qiagen DNeasy Blood and Tissue Kit), and different methods of collecting the samples (self-collected with flocked swabs in UTM versus physician collected samples with 10 μl plastic loops). For most species, the threshold values in the present study were 10- to 50-fold higher than those found in the Swedish patients. However, some notable differences were observed; BVAB3 had a 7-fold lower threshold in the women from Greenland, while *Eggerthella*-like bacterium, BVAB1, and *Mobiluncus mulieris* had thresholds approximately 2000-fold higher in the women from Greenland, suggesting a much higher level of carriage in women without BV as determined by Nugent score. Whether these differences reflect true population differences or can be traced back to technical differences remains unclear, but these results emphasise that relevant thresholds should be determined in the population under study using standardised techniques. The vaginal swab specimens were kept in Copan UTM transport medium and refrigerated for up to 5 days before shipment at ambient temperature, typically reaching the laboratory within 1 to 2 days. It cannot be excluded that some growth or even degradation of microbial DNA may have occurred during this time and led to the differences in threshold values. However, the UTM medium contains colistin, vancomycin, and amphotericin B, which restrict the growth of Gram-positive bacteria, Gram-negative bacteria, and fungi, respectively, and because many of the BV-associated bacteria have been excessively difficult to cultivate, distortion of the microflora during transport is most likely insignificant. Furthermore, in another study (Datcu et al., accepted for publication), the bacterial load of eight selected BV associated bacteria in first void urine samples transported in GeneLock (a transport medium designed to stabilise DNA and RNA) was shown to correlate significantly with the bacterial load in the corresponding vaginal swab specimens described in the present study, suggesting that degradation of DNA in UTM during transport is not of major importance.

Using quantitative detection and Nugent scoring as the gold standard for BV, the combination of *Prevotella* spp. *and A. vaginae* had a high specificity at 99%, but the sensitivity decreased to 90%. When considering *Prevotella* spp. *or A. vaginae*, the specificity decreased to 89% while the sensitivity increased to 100%. Thus, by combining “*Prevotella* spp. *and/or A. vaginae*,” it is possible to select either an almost perfect specificity or an optimal sensitivity. Consequently, these 2 real-time PCRs together can diagnose BV with extremely high accuracy.

The average number of non-*Lactobacillus* species represented in the samples, i.e., the diversity, increased with progression from the normal microbiome (average 6.2 species with a range of 3–10) to BV (average 11.7 species, range 5–16). Fredricks *et al.*[27] found an average of only 3.6 species (range 0–14) detected in women without BV, a difference that could be explained partly because PCR assays for *M. hominis*, *U. urealyticum,* and *U. parvum* were not performed and one PCR assay was used for both *Leptotrichia*/*Sneathia* spp. Compared with data presented for the vaginal flora of US women [27], the flora in women from Greenland was different largely in the detection of nearly all evaluated species in a markedly higher proportion of the women from Greenland with normal microbiota as determined by Nugent’s criteria. However, Zozaya-Hinchliffe [29] found an average of 9.5 non-*Lactobacillus* species for women with normal vaginal flora in samples from US women who attended an STD clinic. Whether this increase reflects differences in the detection limit of the assays or a higher rate of colonisation with these bacteria due to either behavioral or genetic differences remains to be established. The women included in the present study had a high number of lifetime sexual partners, and a previous study by Fethers and colleagues [42] showed that increasing frequency of six of the eight BV candidate organisms included in the study (*Megasphaera* type 1, BVAB 2, BVAB 3, *Sneathia* spp., *Leptotrichia* spp., and *G. vaginalis*) correlated with increasing sexual exposure, suggesting potential sexual transmission of these bacteria.

Using quantitative detection with cut-offs determined by ROC analysis, all non-*Lactobacillus* species except *U. parvum* and *U. urealyticum* were significantly associated with BV in univariate analysis. *Prevotella* spp. and *A. vaginae* remained significantly associated with BV in a multivariate logistic regression model performed on the seven species selected by their AUC values of > 85% in ROC analysis.

The women with Nugent grade III could be further divided into distinct sub-types of BV flora dominated by different bacterial species, such as *Prevotella* spp., *G. vaginalis*, or BVAB 1, or by combinations of species, dominated by *G. vaginalis*/*Prevotella* spp. and BVAB1/*G. vaginalis*. This finding is in accordance to some extent with other studies using 454 pyrosequencing of amplicons generated with universal 16S primers [43], although the clusters identified in the present study were somewhat different, particularly with *Sneathia/Leptotrichia* spp*.*-dominated clusters being less prominent. Whether this difference reflects methodological or true population-dependent differences is not clear*.* The sub-clustering clearly suggests that the diagnosis of BV may be a simplified grouping of several conditions and refined diagnostic methods may prove useful in both stratifying the treatment of the condition and targeting treatment to prevent complications in relevant subgroups.

*Prevotella* spp. have been found in the majority of patients in culture-based surveys of vaginal microbiomes [44] and are one of the most common operational taxonomic units (OTUs) present in recent molecular studies [45]. *Prevotella* spp. and BVAB1 have been reported to dominate in single taxon clusters of BV communities [43]. Bacteria that dominate in pairs may act synergistically or may have a symbiotic or even mutual relationship; this may be the case for *G. vaginalis* with *Prevotella* spp.; it has been shown that amino acids produced during *G. vaginalis* growth may promote the growth of *P. bivia*. Conversely, the growth of *G. vaginalis* becomes limited by a shortage in available ammonia, which can be provided by *P. bivia*[46]. However, we and others have found that *G. vaginalis* dominated in BV clusters by itself, in the absence of high concentrations of *Prevotella* spp., suggesting that there may be other mechanisms not clearly understood to sustain the growth of this bacterium.

Correlations between species were also found. Most notably, BVAB2 had strong correlations with *A. vaginae*, *S. sanguinegens*, *L. amnionii*, BVAB3, *Megasphaera* type 1, and *Eggerthella-*like bacteria*.* This is in agreement with findings using 454 pyrosequencing [43], although this study found positive correlations with some *Prevotella* species but negative correlations with others. However, the less discriminative approach chosen in the present study can easily explain this discrepancy.

The lack of specific detection of other *Lactobacillus* spp. reported to be part of the normal microbiome of the human vagina, such as *Lactobacillus crispatus*, *Lactobacillus jensenii*, and *Lactobacillus gasseri*, is a potential limitation of the study presented here. However, as these species are mainly associated with healthy vaginal flora, the conclusions reached for women with BV should not be affected. Although using specific quantitative PCRs limits the study to selected species, a major strength of this approach is that individually performed assays are less susceptible to amplification bias. In broad-range 16S PCR, differences in the amplification efficiency resulting from imperfect sequence matches of the primers with some species as well as competitive amplification in which the most abundant species tend to dominate may skew the species distribution. Future research should determine which sub-types of BV communities confer significant health risks to women and aim to stratify treatment to avoid the common recurrence of BV.

## Conclusions

In summary, our study confirmed a high prevalence of BV in women from Greenland. *A. vaginae* and *G. vaginalis* were present in all patients with BV by qualitative detection and also in a high proportion of women with normal microbiota as determined by Nugent’s criteria. Using cut-offs determined by ROC curve analysis and a combination of *Prevotella* spp. and/or *A. vaginae* PCR, we were able to diagnose BV with high accuracy. *Prevotella* spp. had the highest odds-ratio and adjusted odds-ratio for BV in univariate analysis. In multivariate logistic analysis, *Prevotella* spp. and *A. vaginae* were both independently associated with BV. Different clusters of BV communities could be identified in women from Greenland, some of which were dominated by one or two bacterial species, suggesting metabolic co-dependencies. Further molecular studies are needed to establish the clinical consequences of the different BV profiles.

## Competing interests

The authors declared that they have no competing interests.

## Authors’ contributions

Conceived and designed the experiments: JSJ, PA, RD, DG, ER. Performed the experiments: RD. Analysed the data: RD, JSJ, PA. Contributed reagents/materials/analysis tools: JSJ, PA, RD, DG, GM, RMA, ER, AK. Wrote the manuscript: RD, PA, JSJ. All authors read and approved the final version of the manuscript.

## Pre-publication history

The pre-publication history for this paper can be accessed here:

http://www.biomedcentral.com/1471-2334/13/480/prepub

## Supplementary Material

Additional file 1: Table S1 PCR primers and probes with corresponding product sizes, annealing temperatures, and Mg^++^ concentrations. Click here for file

Additional file 2: Table S2 Spearman correlation coefficients among bacterial species in swabs showing co-occurrence of species. Click here for file
