# Supplementary material for: Vaginal microbiome in women from Greenland assessed by microscopy and quantitative PCR
Source: BMC Infect Dis. 2013 Oct 16;13:480. doi: 10.1186/1471-2334-13-480 (PMC3853076; doi:10.1186/1471-2334-13-480)
Supplement: Additional file 1: Table S1 — PCR primers and probes with corresponding product sizes, annealing temperatures, and Mg++ concentrations. [file 1471-2334-13-480-S1.docx]

**Table S1 (Additional file 1).** PCR primers and probes with corresponding product size, annealing temperature, and Mg^++^ concentration.

| Microorganism  (Genbank no.) | Forward primer (5’-3’) | Reverse primer (5’-3’) | TaqMan® probe (5’-3’) | Product size (bp) | Ta (°C) | MgCl2  final conc.(mM) | Limit of detection^1^  (copies/ml UTM) |
| --- | --- | --- | --- | --- | --- | --- | --- |
| *Atopobium vaginae* [NR_029349] | CGGTCTGTTAGGTCAGGAGTTAAATC | CCATCTTCCCCTACCAGACTCA | FAM-CTCAACCCCTATCCGCTCCTGATACCG  BHQ-1 | 88 | 60 | 5 | 32 |
| *Sneathia sanguinegenes* [NR_025487] | GACTGGGATAACAGRGGGAAACTTC | TCATATAGCGTATTGCTACCTTTCATTAC | VIC- CTGGATAAGTTAGTAGTAGCATTA- MGB | 90 | 60 | 5 | 48 |
| *Leptorichia amnionii* [EF218612] | GACTGGGATAACAGRGGGAAACTTC | GGACGCAAAGCTCTCCTTTAGTG | FAM- ATAAGTTAGTATATCGCATGATAT-MGB | 101 | 60 | 5 | 16 |
| BVAB1 [AY724739] | **GTATATTTTCTACGGAACACAGG** [27] | **~~T~~TTGCTCCGGATCGCTCCTT** [27] |  | 260 | 55 | 2.5 | 127 |
| BVAB2 [AY724740] | GC**TTAACCTTGGGGTTCATTACAA** [28] | CG**GAATACTTATTGTGTTAACTGCGC** [28] |  | 265 | 55 | 2.5 | 32 |
| BVAB3 [AY724741] | **CCCTTGAACGATGTAGAGATACATAA** [29] | **~~ACATT~~TGGGGATTTGCTTCGCC** [28] |  | 288 | 55 | 1.5 | 16 |
| *Megasphaera* type 1 [AY738672] | **GATGCCAACAGTATCCGTCCG** [27, 28] | **CCTCTCCGACACTCAAGTTCGA** [27, 28] |  | 208 | 55 | 2 | 16 |
| *Megasphaera* type 2 [AY738697] | C**AAGGTGGTAAATAGCCATCATGAG** [27] | TTC**CTCTCCGACACTCAAGTCTTC** [27] |  | 214 | 55 | 3.5 | 48 |
| *Eggerthella-* like bacterium [AY738656] | **AACCTCGAGCCGGGTTCC** [27] | **TCGGCACGGAAGATGTAATCT** [27] |  | 236 | 55 | 2.5 | 48 |
| BVAB TM7 [AY738690] | G**AACTGCTTGGCTCGAGATTATC** [27] | TC**TCTCCTTTCGGAGAAATTCTAGG** [27] |  | 385 | 55 | 3.5 | 2698 |
| *Mobiluncus curtisii* [AJ318408] | **TTCTCGCGAAAAAGGCACAG** [27] | **~~C~~TGGCCCATCTCTGGAACCA** [27] |  | 581 | 60 | 2 | 794 |
| *Mobiluncus mulieris* [AJ576087] | **~~G~~CTCGTAGGTGGTTCGTCGC** [27] | G**CCACACCATCTCTGGCATG** [27] |  | 452 | 60 | 1.5 | 4444 |
| *Lactobacillus iners* [Y16329] | CGA**GTCTGCCTTGAAGATCGG** [22] | G**TTATCCCGATCTCTTGGGCA** [29] | FAM- CTTGCACTCTGTGAAACAAGATACAGGCTAGC -BHQ | 108 | 60 | 5 | 111 |
| *Prevotella spp.* [JN867318] | **~~G~~GGATGCGTCTGATTAGCTTGTT** [29] | **~~CT~~GCACGCTACTTGGCTGGTTC** [29] |  | 176 | 55 | 1.5 | 317 |
| *Gardnerella* *vaginalis* [AY738668] | **ACCTGGGCTTGACATGTGCCT** [28] | **CATGCACCACCTGTGAACCTG** [28] | **FAM-CTGCAGAGATGTGGTTTCCYTTCG-~~TAMRA~~-**BHQ [28] | 74 | 60 | 5 | 317 |
| *Mycoplasma hominis* [M24473] | CATGCATGTCGAGCGAGGTT | CCATGCGGTTCCATGCGT | FAM- CATTGTTTCCAATGGGT-MGB | 129 | 60 | 5 | 32 |
| *Ureaplasma parvum* | GCAAGAAGACGTTTAGCTAGAGGTTT | CGAGCAGATTGCATTAGGTCAG | VIC-TTTAATTACTGATCATGTAATGGA-MGB | 124 | 60 | 5 | 16 |
| *Ureaplasma urealyticum* | GCAAGAAGACGTTTAGCTAGAGGTTT | CACGAGCAGATTGCATTAAGTCAG | FAM-TAATTACTGACCACGTAGTGGA-MGB | 126 | 60 | 5 | 95 |
| *Finegoldia magna* [D14149] | GACAACCTGCCTATGACAGTG | AGAAAGTCGCCTTCGCTACT |  | 578 | 55 | 1.5 | 159 |
| *Mycoplasma genitalium* [M31431] | **GAGAAATACCTTGATGGTCAGCAA** [21] | **GTTAATATCATATAAAGCTCTACCGTTGTTATC** [21] | **FAM**-**ACTTTGCAATCAGAAGGT**-**MGB** [21] | 78 | 60 | 5 | NA^2^ |
| *Mycoplasma genitalium* [NR_026155] | **TACATGCAAGTCGATCGGAAGTAGC** [23] | **AAACTCCAGCCATTGCCTGCTAG** [23] |  | 425 | 62 | 2.5 | NA^2^ |
| *Chlamydia trachomatis* [DQ019310] | **GGATCTTAGGACCTTTCGGT** [24] | **ATCTCTCAATCCGCCTAGACG** [24] | **TET-AAGGGAGAG TCTATGTGATAT**-**MGB** [24] | 101 | 60 | 5 | NA^2^ |
| *Chlamydia trachomatis* [X06707] | **GGATCCGTAAGTTAGACG AAATTTTG** [24] | **TTTAATGCGAAAGGAAATCTGATTG** [24] | **Yakima Yellow-TTTGCGCACAGACGATCTATTTTTTGCA-BHQ-1** [24] | 83 | 60 | 5 | NA^2^ |
| *Neisseria gonorrhoeae* [AJ223449] | **GTTTCAGCGGCAGCATTCA** [25] | **CCGGAACTGGTTTCATCTGATT** [25] | FAM-CAGCAAGTCCGCCTATAC GCCTGCTACTT-BHQ-1 | 102 | 60 | 5 | NA^2^ |
| *Trichomonas vaginalis* [L23861] | **ATTGTCGAACATTGGTCTTACCCTC** [26] | **TCTGTGCCGTCTTCAAGTATGC** [26] |  | 262 | 60 | 4 | NA^2^ |

Sequences in bold correspond to published primers. For primers modified to obtain equal Tm values, strike-through letters indicate deletion of

nucleotides from the published sequence, whereas underlined letters indicate additional sequence.

Primers and/or probes designed for the present study are in regular print.

^1^Limit of detection of the PCR assays was defined as the minimum number of 16S rRNA gene copies/ml UTM that can be detected with the q-PCR assay.

^2^NA: not applicable, as the results of real-time PCR assays were not used quantitatively.
